# Supplementary material for: Coconut Oil Alleviates the Oxidative Stress-Mediated Inflammatory Response via Regulating the MAPK Pathway in Particulate Matter-Stimulated Alveolar Macrophages
Source: Molecules. 2022 May 2;27(9):2898. doi: 10.3390/molecules27092898 (PMC9105152; doi:10.3390/molecules27092898)
Supplement: Supplementary file 1 [file molecules-27-02898-s001.zip › molecules-1684366-supplementary.pdf]

Supplementary information

Coconut oil alleviates the oxidative stress-mediated inflammatory response via regulating MAPK pathway in particulate matter-stimulated alveolar macrophages

Xinyu Chen[1,2], Dong Im Kim[1], Hi-Gyu Moon[1], Minchul Chu[3], and Kyuhong Lee[1,2,*]

[1]Inhalation Toxicology Center for Airborne Risk Factor, Korea Institute of Toxicology, 30 Baehak1-gil, Jeongeup, Jeollabuk-do, 56212, Republic of Korea.; chen.xinyu@kitox.re.kr, dongim.kim@kitox.re.kr, higyu.moon@kitox.re.kr
[2]Department of Human and Environmental Toxicology, University of Science & Technology, Daejeon, 34113, Republic of Korea.
[3]GREENSOL CO., LTD., 89-26, Jimok-ro, Paju-si, Gyeonggi-do, 10880, Republic of Korea.; chumin6329@naver.com
[*]Correspondence: khlee@kitox.re.kr, khleekit@gmail.com; Tel.: +82-63-570-8740

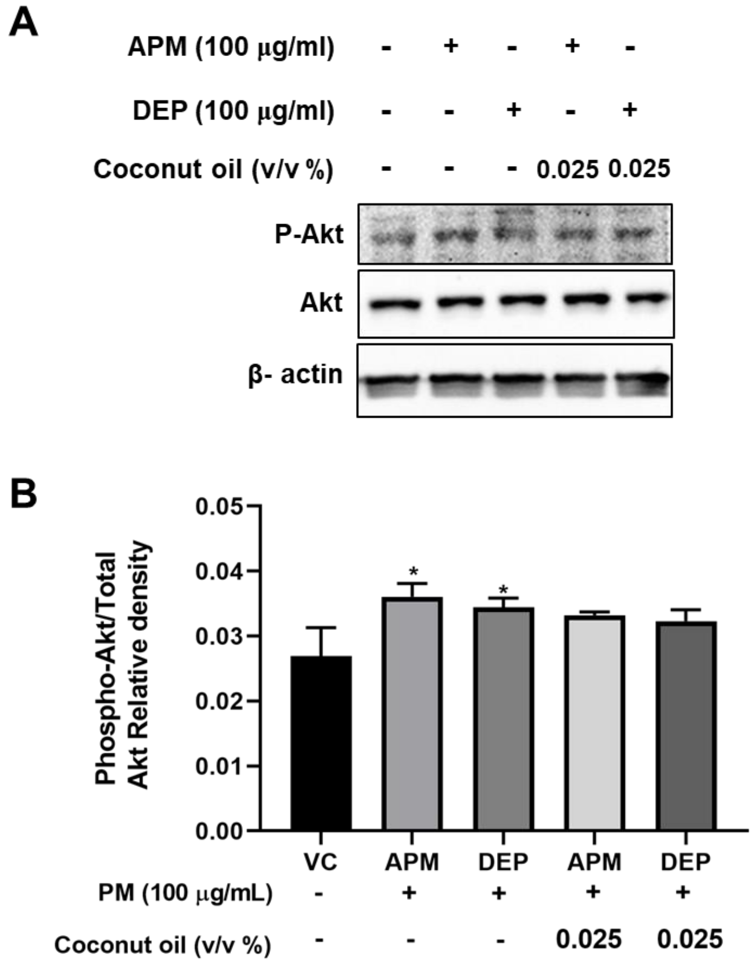

Figure S1. Representative western blots and relative density of phosphorylation of Akt expression in artificial particulate matter (APM)- or diesel exhaust particles (DEP)-stimulated alveolar macrophages (MH-S). Vehicle control (VC) was treated with cell medium including 10% distilled water. MH-S was treated with APM and DEP of different concentration for 6 h. Data are presented as the means ± SD (n = 3 per group). *$p<0.05$ vs. VC.
